# The role of sex and gender in acute kidney injury—consensus statements from the 33rd Acute Disease Quality Initiative

**DOI:** 10.1016/j.kint.2025.01.008

**Published:** 2025-01-21

**Authors:** Danielle E. Soranno, Linda Awdishu, Sean M. Bagshaw, David Basile, Samira Bell, Azra Bihorac, Joseph Bonventre, Alessandra Brendolan, Rolando Claure-Del Granado, David Collister, Lisa M. Curtis, Kristin Dolan, Dana Y. Fuhrman, Zahraa Habeeb, Michael P. Hutchens, Kianoush B. Kashani, Nuttha Lumlertgul, Mignon McCulloch, Shina Menon, Amira Mohamed, Neesh Pannu, Karen Reue, Claudio Ronco, Manisha Sahay, Emily See, Michael Zappitelli, Ravindra Mehta, Marlies Ostermann

**Affiliations:** 1 Department of Pediatrics, Indiana University School of Medicine, Indianapolis, Indiana, USA; 2 Division of Clinical Pharmacy, University of California, San Diego Skaggs School of Pharmacy and Pharmaceutical Sciences, La Jolla, California, USA; 3 Department of Critical Care Medicine, Faculty of Medicine and Dentistry, University of Alberta, Edmonton, Alberta, Canada; 4 Department of Anatomy Cell Biology and Physiology, Indiana University School of Medicine, Indianapolis, Indiana, USA; 5 Division of Population Health and Genomics, University of Dundee, Dundee, UK; 6 University of Florida, Gainesville, Florida, USA; 7 Department of Medicine, Harvard Medical School, Boston, Massachusetts, USA; 8 International Renal Research Institute of Vicenza, Vicenza, Italy; 9 Division of Nephrology, Department of Medicine, Hospital Obrero No 2 Caja Nacional de Salud, Cochabamba, Bolivia; 10 Division of Nephrology, Department of Medicine, Faculty of Medicine and Dentistry, University of Alberta, Edmonton, Alberta, Canada; 11 Division of Nephrology, Department of Medicine, University of Alabama at Birmingham, Birmingham, Alabama, USA; 12 Department of Pediatrics, Texas Children’s Hospital, Houston, Texas, USA; 13 UPMC Department of Pediatrics, Pittsburgh, Pennsylvania, USA; 14 Department of Anesthesiology, Oregon Health & Science University, Portland, Oregon, USA; 15 Division of Nephrology and Hypertension, Department of Medicine, Mayo Clinic, Rochester, Minnesota, USA; 16 Division of Pulmonary and Critical Care Medicine, Department of Medicine, Mayo Clinic, Rochester, Minnesota, USA; 17 Excellence Centre for Critical Care Nephrology, Division of Nephrology, Faculty of Medicine, King Chulalongkorn Memorial Hospital, Bangkok, Thailand; 18 Department of Paediatric Nephrology, Red Cross War Memorial Children’s Hospital, University of Cape Town, Cape Town, South Africa; 19 Division of Nephrology, Department of Pediatrics, Stanford University, Palo Alto, California, USA; 20 Division of Critical Care Medicine, Montefiore Medical Center, Bronx, New York, USA; 21 Department of Human Genetics, David Geffen School of Medicine, University of California, Los Angeles, California, USA; 22 Osmania Medical College and Hospital, Kaloji University of Health Sciences, Hyderabad, Telangana, India; 23 Department of Intensive Care, Royal Melbourne Hospital, Parkville, Victoria, Australia; 24 Hospital for Sick Children, University of Toronto, Toronto, Ontario, Canada; 25 Department of Medicine, University of California San Diego Medical Center, San Diego, California, USA; 26 Department of Critical Care, King’s College London, Guy’s & St Thomas’ Hospital, London, UK

**Keywords:** acute kidney injury, gender, sex differences, social determinants of health

## Abstract

Sex differences exist in acute kidney injury (AKI), and the role that sex and gender play along the AKI care continuum remains unclear. The 33rd Acute Disease Quality Initiative meeting evaluated available data on the role of sex and gender in AKI and identified knowledge gaps. Data from experimental models, pathophysiology, epidemiology, clinical care, gender, social determinants of health, education, and advocacy were reviewed. Recommendations include incorporating sex and gender into research along the bench-to-bedside spectrum; analyzing sex-stratified results; evaluating the effects of sex chromosomes, hormones, and gender on outcomes; considering fluctuations of hormone levels; studying the impact gender may have on access to care; and developing educational tools to inform patients, providers, and stakeholders. This meeting report summarizes what is known about sex and gender along the AKI care continuum and proposes an agenda for translational discovery to elucidate the role of sex and gender in AKI across the lifespan.

Acute kidney injury (AKI) occurs commonly and is associated with morbidity and mortality.^1^ Experimental models have focused on the role of sex hormones and demonstrate that males are more susceptible to AKI.^2–4^ These known sex biases have been used to rationalize single-sex animal models.^5^ Clinical studies have been less clear. The 2012 Kidney Disease: Improving Global Outcomes (KDIGO) AKI guideline listed female sex as a risk factor for AKI, yet numerous studies have demonstrated otherwise.^6,7^ Most clinical and epidemiologic studies do not include sex-stratified analyses and typically encompass broad age ranges that cross puberty and menopause. The potential role of andropause is similarly discounted. We aimed to explore the existing data on the role of sex and gender in AKI, identify gaps in knowledge, and develop consensus statements for management and future research.

## METHODS

The 33rd Acute Disease Quality Initiative (ADQI) meeting was held from March 9 to 11, 2024, in San Diego, CA, and followed the established modified Delphi process described by ADQI.^8^ Working groups were established to review and summarize existing literature up to December 2023 and identify key questions with respect to sex and gender in AKI. Data from experimental studies, epidemiology and pathophysiology in clinical studies, clinical management, the role of social determinants of health (SDoHs), and education and advocacy were appraised.

Before the meeting, each group met virtually 3 to 5 times. During the meeting, each group presented its key questions, proposed consensus statements and recommendations, and received feedback from the larger panel in an iterative fashion until consensus was reached. The Grading of Recommendations, Assessment, Development and Evaluation (GRADE) system was used to rate the evidence of recommendations. Where evidence was lacking, recommendations for future research were generated. Consensus was determined by an affirmative vote of >85%.

Many, if not most, clinical studies conflate sex with gender. Sex is a biological variable, and gender is a societal construct. Although gender and sex are aligned in most patients, we recognize that there are patients in whom gender presentation does not align with biological sex. Sociocultural norms may impact this prevalence and vary across the world. The gender-diverse/transgender population accounts for 0.5% to 3% in some regions and has unique kidney health and disease considerations. However, these considerations are outside the scope of our recommendations.

## RESULTS

### Pathophysiology of sex biases

#### Question (Q) 1: What sex differences have been identified in experimental model systems?

In experimental model systems, males are usually more susceptible to AKI.

#### Q2: What are the mechanisms that underlie sex differences in the development, severity, recovery, and sequelae of AKI, acute kidney disease, and transition to chronic kidney disease (CKD)?

Variations in vascular, inflammatory, antioxidant, and other cytoprotective pathways that modify AKI result from sex differences in baseline physiology and response to injury.

#### Q3: How do sex differences in nonkidney diseases and physiological states influence the risk and pathology of AKI in males and females?

The impact of sex on organ crosstalk and AKI is not well understood.

### Rationale

The literature converges on the observation that males are more susceptible to ischemia/reperfusion or toxin injury than females, with structural and functional differences.^5^ However, female protection may be lost in aged animals^9^ or in the degree of AKI to CKD transition.^4^ This finding remains controversial as others have demonstrated the opposite. Thus, it highlights the need for further exploration of the role of hormonal changes across the lifespan.^10,11^ Extensive literature supports the idea that gonadal hormones underlie sex differences. Generally speaking, male kidneys are larger, with proximal tubule hypertrophy, higher mitochondrial content, and differing expression of transporters.^12^ There are sex differences in the relative abundance of transporters along the tubule, which may explain in part the sex differences in risk of AKI after nephrotoxic exposures.^13^ There are vast differences in gene expression in male vs. female kidneys,^12,14^ with alterations in components of metabolic pathways. The biochemical milieu within the female kidney may be similar to protective preconditioning pathways. For example, estrogen stimulates and testosterone impairs superoxide dismutase responses, leading to enhanced oxidative stress in AKI.^15,16^ Heat shock protein 72, a cytoprotective protein induced in cardiac myocytes by estrogen, is present at approximately 40% higher levels in female rat kidneys than in male kidneys.^17^

Hormonal and chromosomal effects drive differences in kidney physiology between males and females. Differences in vascular, inflammatory, antioxidant, and other cytoprotective pathways that modify AKI and its consequences may relate to baseline sex differences or adaptations to injury. Estrogen promotes the stability of the glomerular endothelial barrier in mice in the setting of ischemia/reperfusion AKI.^18^ Estrogen directly promotes, and testosterone impairs, nitric oxide–dependent vasodilation.^19^ Other vasoactive pathways, such as the 12/15 lysyl oxidase pathway^20^ and Cyp4a14, manifest sex differences associated with injury.^21^ Female sex confers relative resistance to inflammation in the setting of AKI. In some inflammatory models, estrogen suppresses the activation of lymphocytes and neutrophils, which are important in the pathogenesis of AKI.^22^ Sex hormone receptor signaling may also play a role in AKI susceptibility. Estrogen receptor-α is associated with protective effects, whereas the increased susceptibility to ischemia-reperfusion AKI in males has been shown to be independent of androgen receptors and dependent on testosterone.^2,16,23^

The influence of chromosomal sex (XX or XY) on AKI is not well characterized, but in rat chromosome substitution strains, the X chromosome from an AKI-resistant strain was protective when introgressed into an AKI-susceptible genetic background.^24^ Data suggesting that sex chromosome complement influences injury sustained after ischemia/reperfusion in the heart and brain^25^ raise the possibility that similar effects may occur in AKI and can be studied using mouse models.^26^ Experimental models can also be used to investigate the impact of incomplete X chromosome inactivation, which results in an imbalance of X chromosome dosing of certain genes in females compared with males, the rate of which varies across species.^27^

**Figure 1 |:**
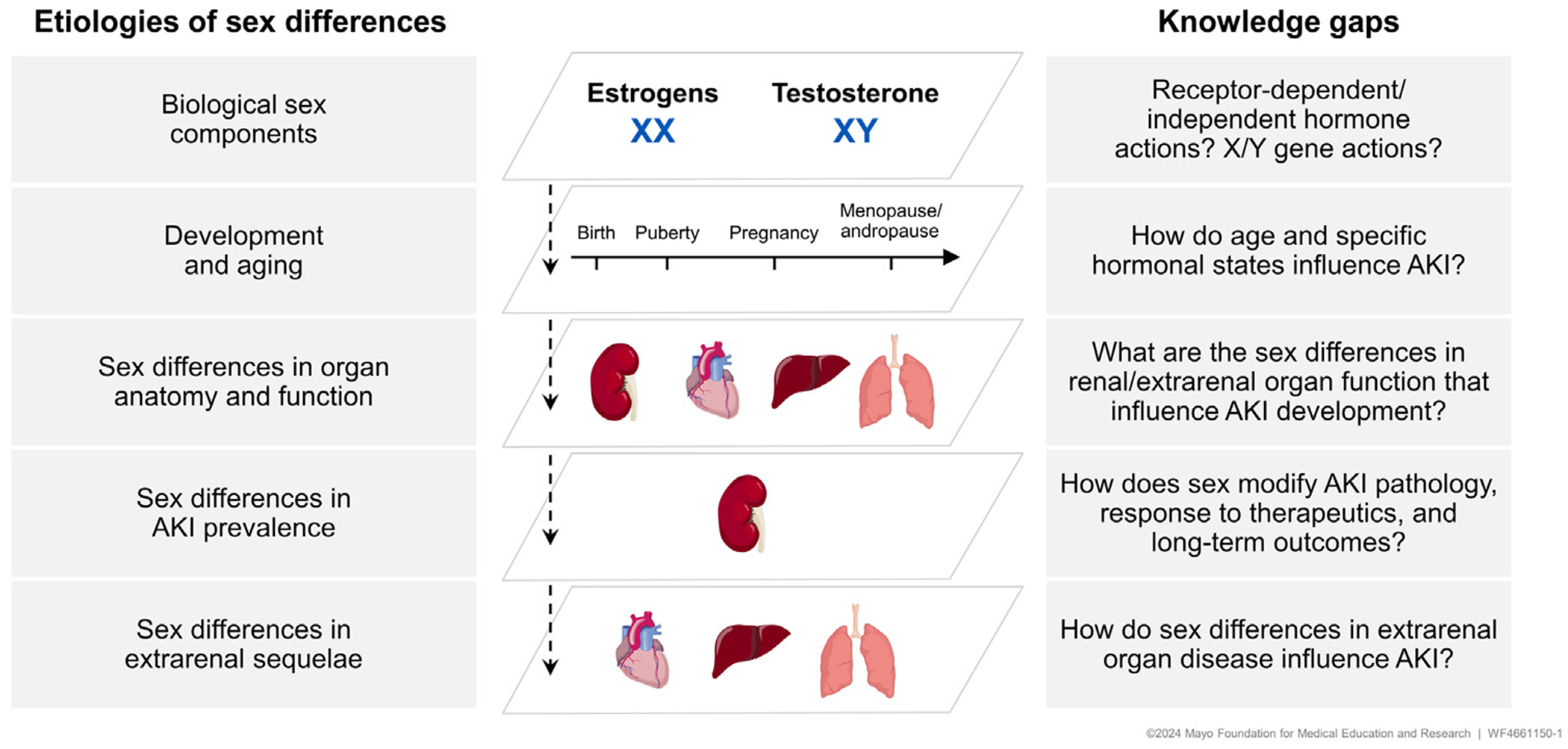
Key aspects of sex differences in animal modeling of acute kidney injury (AKI). Hormones and chromosomes underpin many of the phenotypic and physiological aspects of sex. Differences throughout lifespan may impart hormonal influences that impact sex effects, from birth and early development through menopause and andropause to old age. Sex differences in AKI and its long-term sequelae may be influenced by interactions with other organ systems, by extrarenal diseases such as heart or liver disease, or by physiological states such as pregnancy. © 2024 Mayo Foundation for Educational Research.

The study of extrarenal organ influence on AKI is limited to acute lung injury, sepsis, and acute cardiorenal syndrome. Mechanical ventilation affects kidney function, and acute lung injury induces kidney injury.^28^ Although sex differences in acute lung injury are controversial, rodent models demonstrate estrogen-mediated female protection.^29,30^ Rodent studies demonstrate that acute cardiorenal syndrome is worse in estrogen-deprived females and males.^31^ Rodent models of cardiac surgery exist but have not interrogated sex differences.^32^ Although studies have shown that hemodilution during cardiopulmonary bypass is a risk factor for AKI, others have shown that women may not have a higher risk of AKI than men despite having greater hemodilution.^33,34^ Additional studies in crosstalk models evaluating mechanisms of sex differences are required to delineate mechanisms of differences in risk of AKI and its consequences (Figure 1).

### Epidemiology of sex biases and considerations for clinical research

#### Q4. What is known about the influence of sex differences on AKI in clinical and epidemiologic research?

There are sex differences in the susceptibility, risk, and diagnosis of AKI and associated outcomes, which may bias eligibility and enrollment for participation in research. The effect of changes in sex hormones on the modification of AKI risk and outcome is not well described.

#### Q5. What is known about the influence of sex on differences in the receipt, response to, and outcomes of AKI therapies?

There are sex differences in the receipt of therapies to prevent AKI. Sex differences in anatomy, body composition and size, and pharmacokinetics/pharmacodynamics may influence the receipt of, response to, and outcome of AKI therapies.

#### Q6. How are sex and/or gender considered in the design, conduct, analysis, and reporting of clinical and epidemiologic AKI research?

Sex and gender are often used interchangeably. Females/women are under-represented. Reporting of sex- or gender-stratified results is infrequent.

### Rationale

AKI susceptibility may be impacted by sex differences in the incidence and severity of comorbidities associated with AKI, whereas AKI risk can be directly altered by social behaviors, biological factors, and sex-specific therapies.^35^ Sex differences in nephron mass, creatinine generation/elimination, and care processes influence baseline creatinine and AKI diagnosis.^36^ This may subsequently bias eligibility and enrollment in epidemiologic research and clinical trials. It is unknown whether methods commonly used to impute missing data in AKI research are equally valid in women and men.

**Figure 2 |:**
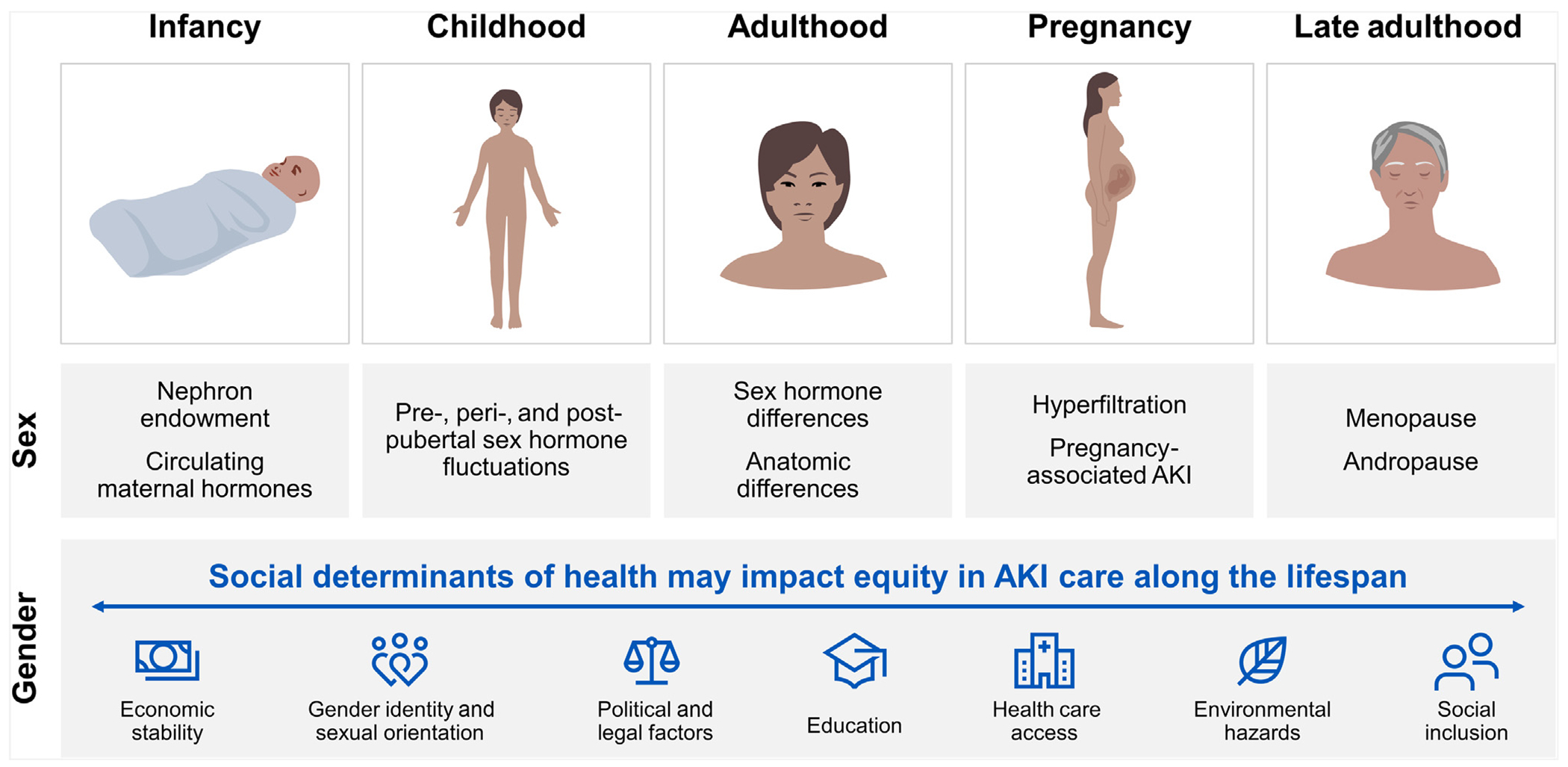
The role of sex and gender on acute kidney injury (AKI) care along the lifespan. Sex and gender differences can impact patients along the AKI care continuum throughout their lifespan. Sex is a biological variable, and differences between males and females can be caused by differences in sex hormones or chromosomes. Gender is a societal construct, along with other social determinants of health, and may impact care at any time throughout a patient’s lifespan.

The specific physiological and pathophysiological mechanisms by which sex hormones (and their changes across the lifespan) impact AKI in humans are not well understood. Based on experimental data, it can be hypothesized that sex hormones influence AKI susceptibility, risk, and diagnosis.^37,38^ Seminal experimental work by Bonventre *et al.*^2,16^ demonstrated that testosterone, more than estrogen, accounted for sex differences in the severity of ischemia-reperfusion AKI via non-androgen receptor mechanisms. However, a clinical study in men with prostate cancer showed a higher risk of AKI in men receiving androgen deprivation therapy.^39,40^ There is a need to develop and validate methods to ascertain the biological, developmental, and acquired sex hormone status (e.g., hormonal contraception, hormone replacement or blocking therapies, and gender-affirming care) of research participants (Figure 2). Studies in postmenopausal women undergoing estrogen replacement therapy and in transgender individuals indicate that estrogen has renoprotective effects in AKI.^41,42^ Conflicting studies about sex differences in cardiac surgery–associated AKI are informative;^6^ here, age may be a driving factor^5,43^ because women undergoing cardiac surgery are often older than men.^6^

Sex differences in the receipt of therapies that affect AKI risk have been reported, including the volume of intravenous fluids and type of antibiotics in sepsis.^44^ Anatomic differences may influence empirical AKI management, and differences in body composition and size may impact the comparative “dose” of AKI therapies.^45^ Social behaviors and gender roles may also determine therapies, including the receipt of kidney replacement therapy.^46^

There is a lack of consistency in how sex and gender are reported and described in the AKI literature (Supplementary Table S1). In many studies, terms for sex and gender are used interchangeably, and gender is often used in reference to biological sex. Women are under-represented in clinical kidney trials and have higher attrition after enrollment.^47^ This may reflect previous restrictions on women with childbearing potential from entering clinical trials and the frequent exclusion of sex-related conditions. Representative enrollment of women in trials is critical to ensuring the efficacy and safety of therapies for all patients. Several methods exist to integrate sex-specific differences into the conduct, analysis, and reporting of clinical and epidemiologic research (Table 1). Sex-stratified analyses have been infrequently presented and are rarely of sufficient size or power to derive meaningful conclusions. A systematic review of AKI studies published from 1978 to 2018 identified only 83 studies (of 6984 screened abstracts) that reported sex-stratified data.^7^ More commonly, especially in pediatric AKI studies, sex-adjusted rather than sex-stratified analyses are performed, which may obscure significant differences in efficacy, safety, and tolerability. For example, a recent study demonstrated an increased risk of death in pubertal females with sickle cell–associated AKI compared with males and that outcomes were worse for postpubertal females.^48^

### The role of sex and gender across the 5 Rs of AKI

In response to the need to raise awareness and improve the care of children and adults at risk of, or with AKI, we applied the conceptual 5 Rs approach (risk, recognition, response, kidney replacement therapy, and rehabilitation) with consideration of sex and gender.^49^

#### Q7: How might differential risks for AKI be applied in clinical practice?

We suggest that clinicians be aware that modifiable and nonmodifiable AKI risk factors may differ by sex. *(Strong recommendation, moderate quality of evidence)*

#### Q8: What factors might clinicians consider in the recognition of AKI based on sex?

We suggest consideration of sex differences when interpreting biomarkers to monitor kidney health. *(Conditional recommendation, low quality of evidence)*

#### Q9: How might clinicians individualize therapeutic interventions for AKI based on sex?

We suggest individualizing drug prescribing and monitoring with consideration of sex differences across the lifespan. *(Conditional recommendation, low quality of evidence)*

#### Q10: How can differences among the sexes concerning the prescription, delivery, and monitoring of kidney support therapies be applied in the care of patients?

- We suggest that the decision-making factors regarding initiation and discontinuation of kidney support therapies not differentiate between sexes, apart from pregnancy-related AKI. *(Strong recommendation, moderate quality of evidence)*
- We suggest that clinicians consider sex differences in solute generation rates and distribution volumes in the treatment prescription and delivery of kidney support therapies. *(Weak recommendation, low quality of evidence)*

#### Q11: Is the care after AKI different between sexes?

It is unknown whether care after AKI differs based on sex.

### Rationale

There are sex-based differences in AKI risk factors such as diabetes, heart failure, liver disease, cancer, sickle cell disease, and nephrotoxin exposure. In the general population, lower estimated glomerular filtration rate (eGFR) and higher urinary albumin to creatinine ratio were associated with higher AKI risk in both males and females, but males had a higher AKI risk at all levels of eGFR and urinary albumin to creatinine ratio. In CKD cohorts, male sex was associated with higher AKI risk at eGFR >40 ml/min per 1.73 m^2^ and urinary albumin to creatinine ratio >300 mg/g, and males had higher adjusted incidence rates at all levels of eGFR and urinary albumin to creatinine ratio.^50^ Notably, this and several other studies have relied on International Classification of Diseases codes to report the incidence of AKI. However, it has been demonstrated that International Classification of Diseases codes are more sensitive in detecting AKI in men than women.^51^ Table 2 summarizes risk prediction models for AKI concerning sex and/or gender for clinical practice. Other risk stratification tools exist, such as the one by Kheterpal *et al.*,^62^ which identifies male sex as a risk factor for developing AKI within 30 days of surgery.

Creatinine generation and excretion differ by sex, body size, comorbidities, and environmental exposures.^50^ Extremes of creatinine kinetics may impact the recognition of AKI and the estimation of kidney function. Both human and animal studies have demonstrated baseline sex differences in urinary biomarker excretion. However, data do not currently support the use of separate normative values for males and females.^63,64^

Sex differences in drug pharmacokinetics/pharmacodynamics affect drug absorption and metabolism and, in turn, drug efficacy, tolerability, and safety.^65^ Sex-specific dosing recommendations are absent for most drugs. Pregnancy leads to significant alterations in drug pharmacokinetics, prolonged gastric emptying, changes in cytochrome P450 and uridine diphosphate glucuronosyltransferase metabolism, and increases in GFR, which may require alterations in dosing and therapeutic drug monitoring.^66^

No data support sex differences in the initiation or discontinuation of acute or chronic kidney replacement therapy, except in pregnant patients, for whom data support earlier kidney replacement therapy due to concerns about fetal complications.^67,68^ Multiple investigations in patients receiving maintenance hemodialysis suggest a survival advantage with increased dialysis dose for women but not men (Supplementary Table S2).^69–73^ Daugirdas *et al.*^74^ demonstrated that rescaling the dialysis dose to body surface area rather than Kt/V may explain these differences because women have a lower anthropometric V per unit of surface area than men. Given sex-specific differences in urea distribution volume, underdialysis in women must be avoided.

No data exist to support differences in rehabilitation after AKI based on sex, including kidney function monitoring and blood pressure management.

### Social determinants of health in gender biases

#### Q12: Which SDoHs influence health equity in the AKI care continuum?

Race, ethnicity, education, socioeconomic status, and environment are likely to influence health equity in the AKI care continuum.

#### Q13: Does sex influence health equity in the AKI care continuum?

The influence of sex on health equity in the AKI care continuum has not been systematically evaluated.

#### Q14: How do the SDoHs intersect with gender and access to the AKI care continuum?

Gender roles and identities affect education, employment status, food security, access to health care, and socioeconomic status, and are likely to influence care across the AKI continuum.

### Rationale

SDoHs, including socioeconomic status, access to quality health care, and disproportionate exposure to environmental factors, are known to affect kidney health, with studies showing associations with risk and progression of CKD.^75^ Documented disparities exist in the health care provided to marginalized communities, particularly in lower-resourced settings, where dialysis and transplant facilities are often lacking.^76^ Inequities persist in access to these vital treatments in high-income countries as well, where access may be influenced by factors such as race, ethnicity, insurance coverage, and immigration status. This results in a focus on acute rather than preventative care.^77^ Lack of awareness among health care providers and culturally competent educational resources may exacerbate inequities in access to care.^75^ The majority of these data are limited to CKD, but it is reasonable that SDoHs also influence AKI care.

Although sex differences in the incidence and outcomes of AKI are recognized, there are limited studies examining their effects on access to care in AKI. Sex differences may influence health equity through traditional gender roles and disparities in gender identities and account for variations in health care–seeking behavior, underscoring the importance of tailored interventions, education, and policies addressing unique needs and challenges.

In addition to often having a lower socioeconomic status and fewer opportunities for education and employment, cultural beliefs in certain countries limit access to health care for women.^75^ Understanding the intersectionality of factors impacting AKI care and identifying intervention strategies is crucial for promoting health equity (Supplementary Figure S1).

### Workforce and patient education

#### Q15: What is needed to raise awareness about sex and gender in the field of AKI?

Advocacy focused on sex and gender is essential to advance awareness and education in AKI. We suggest including sex and gender bias training in routine clinical practice. *(Strong recommendation, moderate quality of evidence)*

#### Q16: What is required to implement education and promote the understanding of sex and gender bias in the care of AKI?

Engaging key stakeholders when creating educational modalities and platforms is essential to improve the knowledge and awareness of the importance of sex and gender in AKI. The identification of advocacy champions may facilitate implementation.

#### Q17: What key performance indicators are required to assess the successful incorporation of sex and gender considerations in education, research, clinical practices, public health, and policy?

Quality indicators focused on sex and gender in AKI require development and validation. These indicators would ideally integrate themes across AKI-focused care structures, processes, and outcomes.

### Rationale

Inclusion of sex and gender is critical to enhancing research relevance and patient care.^5^ As outlined earlier, there are substantial differences in AKI care among the sexes at all levels.^78^ One significant factor in the observed discrepancy in AKI care is poor awareness regarding the influences of sex and gender not only among researchers but also among clinicians, patients, health care systems, and governments.

Primary stakeholders include members of the public representing patients, health care professionals and scientific workforce, health care systems, nongovernmental organizations, industry, and populations (Supplementary Figure S2). Training of members of each stakeholder as educatees and educators should be considered simultaneously or in tandem. Identifying the most efficient modality for stakeholders could enhance their influence on raising awareness.

Stakeholder-specific educational modules must be prepared and implemented (Supplementary Figure S3).^79^ For example, raising sex and gender awareness should include materials that are different for clinicians, patients, policy-makers, and government officials with appropriate platforms (e.g., social media platforms for sex and gender advocacy among younger generations and traditional tools for health care system leaders and politicians).

After the development and implementation of these advocacy programs, it is essential to ensure safe environments for learning and that education evolves based on the current needs assessments. Using a harmonized set of quality metrics in sex and gender advocacy permits comparisons and improvement by continuous benchmarking.^79,80^ These metrics should include (i) structure indicators or input measures that manifest characteristics of educators and platforms; (ii) process indicators of systems and processes to allow for delivery of timely and relevant information tailored for each stakeholder; (iii) outcome measures of educational modules on targets to reduce sex- and gender-based disparities in clinical care, equitable representation in research, and development and implementation of policies; and (iv) balancing measures that assess unintended consequences of educational modules (e.g., the development of new biases, added costs, and dissatisfaction among educators and educates).

## DISCUSSION

The 33rd ADQI consensus statements on the role of sex and gender in AKI are expert-derived and reflect the current state of knowledge. Table 3 summarizes critical gaps and opportunities for future investigation.

## CONCLUSION

Experimental models have established sex differences in AKI outcomes in the setting of equivalent insult and differences in outcomes after achieving a comparable degree of functional injury. Clinical studies often conflate gender with sex and ignore potential confounding effects of changes in sex hormone levels across the lifespan. Opportunities exist to improve the rigor of preclinical and clinical studies concerning both sex and gender and to improve the care delivered to patients.

## Supplementary Material

Supplementary Material

Supplementary material is available online at www.kidney-international.org.

## Figures and Tables

**Table 1 |:** Recommendations for conducting, reporting, and analysis of research studies with considerations of sex and gender

	Observational studies	Randomized controlled trials
Eligibility and conduct	• Use data sources with the inclusion of both sexes or recognize the inclusion of only 1 sex as a study limitation. • Consider inclusion of understudied groups, for example, pregnancy, neonates, prepubertal and postpubertal adolescents, and patients with chromosomal abnormalities. • Acknowledge that the frequency of serum creatinine measurement may influence the ascertainment of AKI diagnosis.	• Consider the influence of sex on susceptibility, risk, and diagnosis of AKI, which may bias eligibility and participation in research. • Consider stratification by sex for randomization. • Consider gender roles in the recruitment of patients and in the selection of study outcomes. • Do not discriminate against patients’ eligibility for research based on sex or gender roles.
Analysis	• Consider reporting gender roles as a variable in the selection of study outcomes. • Consider sex in the subgroup (e.g., pre- vs. postpubertal or pre- vs. postmenopausal) analyses and as covariates for adjustment. • Perform sex-stratified analyses.	• Consider sex in subgroup (e.g., pre- vs. postpubertal, or pre- vs. postmenopausal) analyses and as covariates for adjustment. • Perform sex-aggregated analyses.
Report	• Use sex and gender terminology appropriately. • Report sex-stratified analyses.	• Use sex and gender terminology appropriately. • Report sex-stratified analyses.

AKI, acute kidney injury.

**Table 2 |:** Risk prediction calculators for AKI and their sex/gender covariates from QxMD.com and MDCalc.com

Author (yr)	Journal	Exposure	Outcome	Sex/gender	OR (95% CI)
Basu (2014)^52^	*Kidney International*	Pediatric intensive care unit	Severe AKI	Sex/gender not included in model	Not applicable
Brown (2008)^53^	*American Heart Journal*	Percutaneous coronary intervention	Serious kidney dysfunction	Female sex	1.38 (0.86–2.22)
Gharaibeh (2017)^54^	*Journal of Bone and Joint Surgery*	Total hip arthroplasty	AKI	Male sex	1.78 (1.19–2.7)
McMahon (2013)^55^	*Journal of the American Medical Association Internal Medicine*	Rhabdomyolysis	KRT or in-hospital mortality	Female sex	Not applicable
Mehran (2004)^56^	*Journal of the American College of Cardiology*	Percutaneous coronary intervention	Contrast-induced nephropathy	Sex/gender not included in model	Not applicable
Mehta (2006)^57^	*Circulation*	Cardiac surgery	Postoperative dialysis	Female gender not included in simplified model	0.83 (0.77–0.90)
Nash (2019)^58^	*Nephrology Dialysis Transplantation*	Nonsteroidal antiinflammatory drugs	AKI or hyperkalemia	Male sex/gender	1.44 (1.23–1.67)
Thakar (2005)^59^	*Journal of the American Society of Nephrology*	Cardiac surgery	ARF that required dialysis	Female gender	0.48 (0.21–0.75)
Tsai (2014)^60^	*Journal of the American Heart Association*	Percutaneous coronary intervention	AKI	Sex/gender not included in model	Not applicable
Woo (2021)^61^	*Kidney360*	Surgery	AKI requiring dialysis or MAKE	Sex/gender not included in model	Not applicable

AKI, acute kidney injury; ARF, acute renal failure; CI, confidence interval; KRT, kidney replacement therapy; MAKE, major adverse kidney events; OR, odds ratio.

**Table 3 |:** Knowledge gaps in understanding the role of sex and gender in AKI

Area	Gap	Potential opportunities
Experimental models	1. Insufficient data are available across animal models to fully understand sex determinants of AKI.	1.Expand animal models of AKI to interrogate the effects of sex. a. During developmental stages from birth to puberty to menopause/andropause to old age b. As driven by chromosomal sex and gonadal hormones c. On extrarenal disease and physiological state, which incites and influences AKI
	2. Consideration of differential injury response to the same stimulus in male vs. female animal models may require insult-comparable and injury-comparable models.	2. Incorporate more encompassing definition of injury levels beyond the use of serum/plasma creatry in different sexes and models. a. Use of tissue injury b. Use of GFR c. Use of urine biomarkers d. Use of inflammatory markers e. Use of metabolic changes
	3. Human AKI occurs in the setting of extrarenal disease; animal models may not model sex difference in coexisting or in citing extrarenal disease or physiological states (such as pregnancy).	3. Develop models that allow for greater translation of findings on how sex effects in AKI may be altered in various physiological or pathophysiological states. a. Interaction with therapeutic approaches, such as effects of the use of oral contraceptives or antiestrogens (e.g., those used in breast cancer treatment) on mechanistic aspects of AKI b. Development of models of AKI that incorporate social determinants of health, such as early life stress or nutrition deprivation c. Use of comorbidities in modeling AKI
Epidemiologic and clinical research studies	1. Infrequent presentation of sex-aggregated results	1. Reporting of sex-aggregated results that consider how sex may modify the receipt, prescription, delivery, tolerability, efficacy, safety, and outcomes of therapies to prevent and treat AKI, including KRT
	2. Inconsistent use of sex and gender terms	2. Consistency in the collection and reporting of terms to describe sex and gender in clinical and epidemiologic research with consideration of whether sex- and/or gender-related factors are thought to modify treatment effect
	3. Under-representation of women and females in AKI research	3a. Consideration of gender roles in recruitment of patients in AKI research and in selection of study outcomes 3b. Representation of people of different sexes and genders in definitive RCTs to ensure generalizability of results 3c. Females of childbearing potential should not be unnecessarily excluded or subjected to unnecessarily stringent contraception requirements, unless teratogenicity is a concern.
Risk, recognition, response, KRT, and rehabilitation	1. Sex-specific dosing recommendations are absent for most drugs.	1. Future work should explore differences in therapeutic drug monitoring strategies based on sex and gender.
	2. There are no data on CKRT clearance differences based on sex and/or gender.	2. Descriptive studies focusing on sex-specific differences in practices, patient, and treatment characteristics in patients receiving acute KRT should be conducted.
	3. Study results have shown that kidney outcomes after AKI may be worse in women, but reports are conflicting.	3. Future work should explore the impact of alternative biomarkers for monitoring kidney health after AKI based on sex and/or gender.
Gender and social determinants of health	Influence of SABV on health equity	1.Epidemiologic and clinical research studies on the influence of various SDoHs on AKI 2. Studies assessing the influence of sex and gender on access to care across the AKI continuum
Workforce and patient education, and advocacy	1. Lack of understanding of knowledge gaps in sex and gender bias among all stakeholder groups	1. Identify baseline knowledge of each stakeholder group
	2. Undefined effective educational modalities for each stakeholder group	2. While considering cultural differences between populations, educational programs with a focus on potential biases and on sex and gender should be developed and implemented.
	3. Lack of comprehensive plan to develop, validate, implement, and control educational modalities designed for each stakeholder group	3. Using education design research to create example educational programs for each specific stakeholder group

AKI, acute kidney injury; CKRT, continuous kidney replacement therapy; GFR, glomerular filtration rate; KRT, kidney replacement therapy; RCT, randomized controlled trial; SABV, sex as a biological variable; SDoH, social determinants of health.
