# Supplementary material for: The role of sex and gender in acute kidney injury—consensus statements from the 33rd Acute Disease Quality Initiative
Source: Kidney Int. Author manuscript; Available in PMC 2026 Jul 6. (PMC13334715; doi:10.1016/j.kint.2025.01.008)
Supplement: Supplementary Material [file NIHMS2175954-supplement-Supplementary_Material.docx]

**Supplemental Table S1. Sex and gender considerations of high-impact articles in critical care nephrology with >30 citations published between 2021-2024** (N=98 studies)

|  | **N (%)** |
| --- | --- |
| **Nomenclature** |  |
| *Sex* | 58 (59.2) |
| *Gender* | 12 (12.2) |
| *Both* | 2 (2.0) |
| *Not reported* | 26 (26.5) |
| **Descriptor** |  |
| *Male/Female* | 80 (81.6) |
| *Men/Women* | 12 (12.2) |
| *Both* | 5 (5.2) |
| *Not reported* | 1 (1.0) |
| **Mean proportion of participants** |  |
| *Male/men* | 63.7 ± 9.4 |
| *Female/women* | 36.9 ± 10.5 |
| **Eligibility** |  |
| *Inclusion criteria* | 2 (Male as AKI risk index, different serum creatinine levels for male/female in inclusion criteria) |
| *Exclusion criteria* | 18 (18.4) (Pregnancy, breastfeeding, impending miscarriage) |
| **Randomization** (n=58) (stratification/minimization) | 3 (5.1) |
| **Analysis** |  |
| *Subgroup analysis* | 6 (6.1) |
| *Covariate adjustment* | 29 (29.6) |

*Platnich J, Kung JY, Romanovsky AS, Ostermann M, Wald R, Pannu N, Bagshaw SM. A Systematic Bibliometric Analysis of High-Impact Articles in Critical Care Nephrology. Blood Purif. 2024;53(4):243-267. doi: 10.1159/000535558. Epub 2023 Dec 5. PMID: 38052181; PMCID: PMC10997269.*

**Supplementary Table S2: Studies Including Gender Differences in the Use and Application of Intermittent Hemodialysis for End-Stage Kidney Disease**

| **Author**  **(year)** | **Study Design** | **N** | **Population** | **Findings** |
| --- | --- | --- | --- | --- |
| Eknoyan et al. (2002) | Retrospective Cohort Study | 1,846 | Adult HD patients at 15 centers | When compared to the standard group, the risk of death in the high-dose group was 19% lower in women and 16% higher in men |
| Port et al. (2004) | Retrospective Cohort | 74,120 | Adult HD patients based on two large databases | In women the RR of mortality in women with a URR >75% as compared to 70-75% was lower, this difference was not present in men |
| Spalding et al. (2008) | Prospective Observational | 328 | Adult HD patients | The Kt/V target of 1.2 corresponded to a range of equivalent Kt/BSA and Kt/Weight^0.67^ each significantly higher in males than females |
| Miller  et al.  (2010) | Retrospective Cohort | 88,153 | Adult HD patients from Da Vita Dialysis Clinics | In non-Hispanic white women, a Kt/V > 1.8 exhibited survival advantage trends |
| Weigert et al. (2020) | Retrospective Cohort | 1,247 | Elderly HD patients at five centers in Portugal and Poland | Body weight-adjusted prescribed blood flow rates were lower and blood volume/kg per session was higher in women; no differences in dialysis adequacy |

Abbreviations: *HD* Hemodialysis; *RR* Relative Risk; *URR* Urea Reduction Ratio; *BSA* Body Surface Area; *eGFR* estimated GFR; *OR* Odds Ratio; *CI* Confidence Interval; Kt/V

**Supplementary Figure S1:**


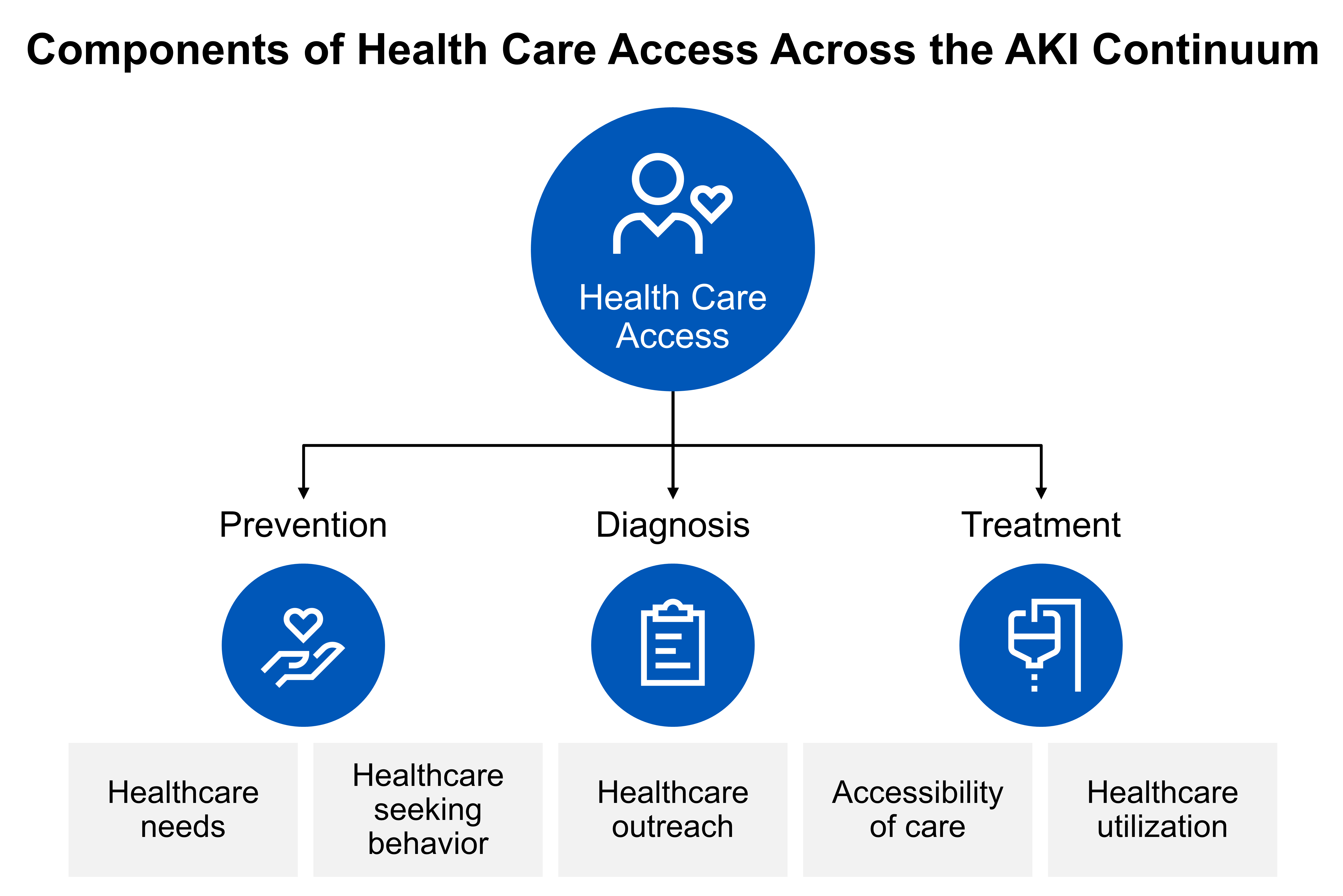


**Supplementary Figure S1. Health care access is a social determinant of health.** Equity with respect to health care access impacts care along the care spectrum, from prevention, to diagnosis, and treatment.

**Supplementary Figure S2:**


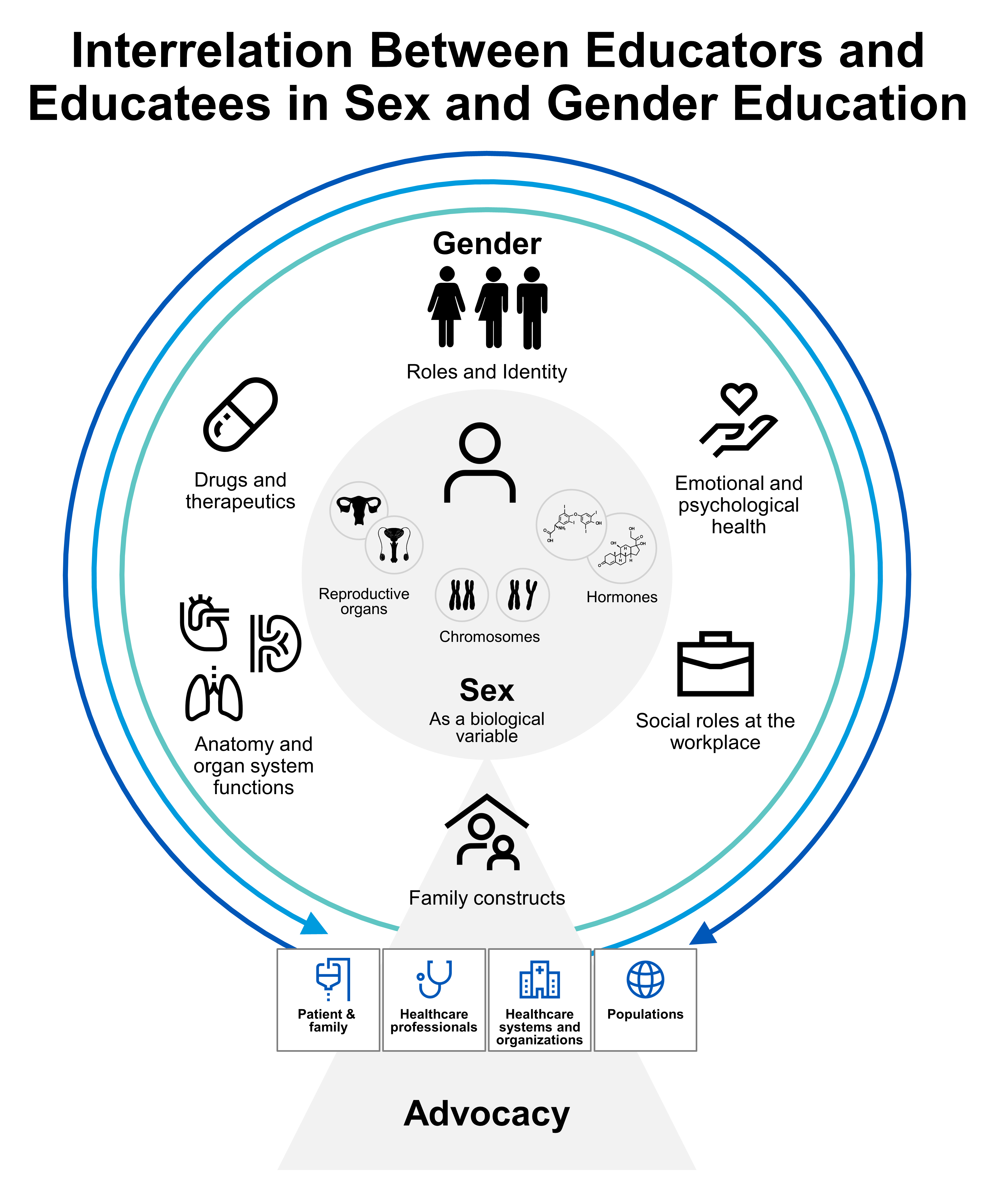


**Supplementary Figure S2. Increasing Awareness of the Role of Sex and Gender on Patient Care.** Educators or educatees are within four domains, including patients/families, healthcare professionals, healthcare systems, and populations. At some point, each of these groups could be educated, and after adequate training, they could become educators and advocates. The education content would focus on both sex as a biological variable and gender as a social construct. In preparing the education content, several factors should be considered, including family construct, workplaces and social role of sex and gender in each society, emotional and psychological health of individuals and populations, and finally, function and organ anatomy, disease processes and their related therapies that could be impacted by sex and gender variability.

**Supplementary Figure S3:**


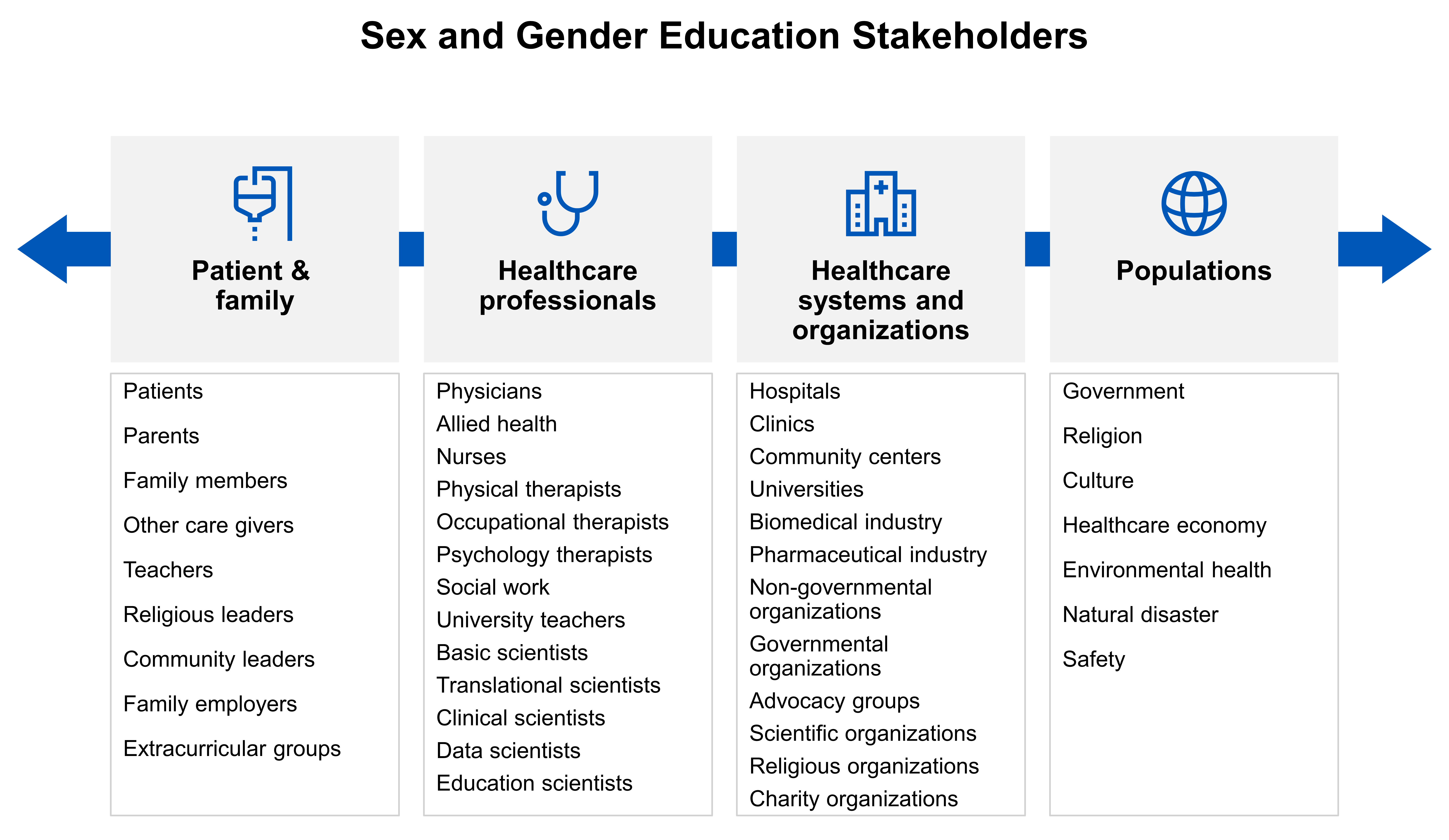


**Supplementary Figure S3. Key stakeholders for education and advocacy.** Education for key stakeholders to increase awareness of the role of sex and gender in care along the AKI continuum requires tailoring efforts toward the specific audience.
